# Storage-Induced Collapse of Lignin Macromolecular Structure and Its Impacts on the Biorefinery

**DOI:** 10.1021/acssuschemeng.5c04284

**Published:** 2025-07-18

**Authors:** Yining Zeng, Kuan-Ting Lin, Renee M. Happs, Juan H. Leal, Xihui Kang, Chang Dou, Jacob S. Kruger, Ling Ding, Kenneth L. Sale, Troy A. Semelsberger, Allison E. Ray, Ning Sun, Bryon S. Donohoe

**Affiliations:** † Renewable Resources and Enabling Sciences Center, 53405 National Renewable Energy Laboratory, Golden, Colorado 80401, United States; ‡ Energy and Environmental Science and Technology, 17212 Idaho National Laboratory, Idaho Falls, Idaho 83415, United States; § Material Physics Applications Division, 5112 Los Alamos National Laboratory, Los Alamos, New Mexico 87545, United States; ∥ Advanced Biofuels and Bioproducts Process Development Unit, Biological Systems and Engineering Division, 1666 Lawrence Berkeley National Laboratory, Berkeley, California 94608, United States; ⊥ Computational Biology and Biophysics, 111651 Sandia National Laboratories, Livermore, California 94500, United States; # Science and Technology, 17212 Idaho National Laboratory, Idaho Falls, Idaho 83415, United States; ∇ Biosciences Center, 53405 National Renewable Energy Laboratory, Golden, Colorado 80401, United States

**Keywords:** lignin, collapse, spectroscopy, microscopy, biorefinery

## Abstract

Lignin plays a vital role in the economics of biorefineries, serving as a source of process energy and a feedstock for sustainable fuels and chemical production. While understanding lignin’s chemical composition is crucial, emerging evidence suggests that a more comprehensive understanding of its macromolecular structure is critical to explaining its complex behavior in the biorefinery. This study investigated the collapse of the lignin network in corn stover feedstock after harvest and storage as a result of the microbial digestion of hemicellulose. Fluorescence microscopy was used to detect the collapse of lignin by the changes in lignin’s fluorescence lifetime, anisotropy, and the number of effective emitters. Our in situ microscopic results revealed lignin’s coil–globule transition phenomena, which was only previously predicted by molecular dynamics modeling of extracted lignin in solvent. This collapse of lignin macromolecular structure was supported by results from NMR, IR, Raman, and powder X-ray diffraction. Our study revealed that the two major approaches for lignin valorization in the lignin-first biorefinery model, namely, monomer extraction and milled wood lignin extraction, were negatively impacted by the lignin collapse. As changes during storage are a source of feedstock variability, our study highlights the importance of understanding the effect of feedstock handling on biorefinery operations and economics.

## Graphical abstract

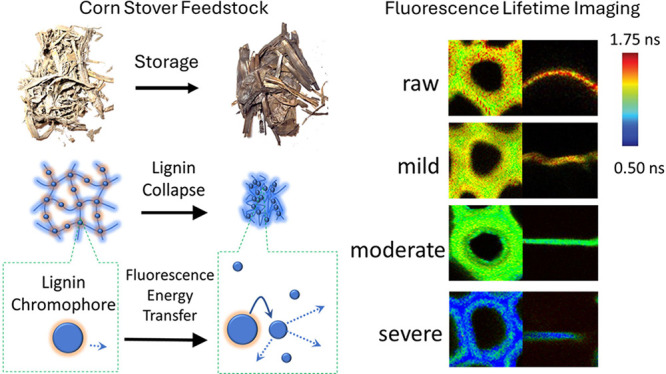

## 1 Introduction

Lignocellulosic biomass is a promising and abundant resource for producing renewable fuels and chemicals.^1^ Lignin, the second most abundant component of lignocellulosic biomass, is a complex and heterogeneous polymer that contributes significantly to biomass recalcitrance.^2^ In addition, recent research has shown that lignin can also be a resource to produce renewable chemicals.^3,4^ Understanding the conformation of the lignin network is important because it provides insights into this critical biopolymer’s role in biorefinery. Storing corn stover feedstock is fundamental to bridging the seasonal harvest windows to supply biorefinery operations. However, understanding of the impact of storage on the lignin network is still limited.

Traditionally, lignin structure has been characterized by many spectroscopic and imaging techniques. Infrared spectroscopy has been used to investigate the functional groups and identify structural changes in lignin, particularly the changes induced by chemical pretreatment processes.^5,6^ X-ray diffraction was applied to determine the crystalline structure of the sample, primarily from crystalline cellulose, and the overall changes in the organization of cell wall polymers were provided.^7,8^ Transmission electron microscopy (TEM), with proper sample staining and labeling, has revealed the cell wall’s architecture and the ultrastructure of lignin.^9^ Nuclear magnetic resonance (NMR) has also been widely applied to analyze the subtle changes in chemical groups in lignocellulosic materials.^10^ However, so far the above spectroscopic (IR, X-ray and NMR) and imaging (TEM) techniques alone have not been able to reveal the macromolecule structural changes of the lignin network involved during the feedstock storage.

Fluorescence microscopy provides spatial resolution high enough to deconvolve the heterogeneity of uneven lignin distribution among cell wall layers. With sensitivity down to single-molecule level, fluorescence microscopy is also decisive for detecting small amounts of compounds in its environment.^11^ In this study, we applied multiple fluorescence microscopic tools to investigate the fluorescence emission of the lignin chromophore in corn stover biomass following field-side storage. We discovered the collapse of the lignin polymer network caused by the biological degradation during storage. The collapse was induced by the reduced intramolecular distance between lignin chromophores, as the hemicellulose was removed by the microbial activities.

The experimentally observed lignin collapse corroborated previous molecular dynamics simulations and spectroscopic studies on extracted cell wall lignins,^12,13^ highlighting the universal occurrence of this collapse not only to isolated lignin but also to lignin in cell wall environments.

Our study found the collapse of the lignin led to a significantly lower yield in lignin alkaline oxidation than theoretically predicted based on the available β-O-4 and ester content. Substantial changes in the structural properties of extracted lignin were also discovered.

## 2 Results

### 2.1 Fluorescence Lifetime Results Revealed the Intramolecular Interactions of Lignin

The corn stover material used for this investigation was harvested in September 2017 in Story County, Iowa, and was stored in uncovered field stacks for two months (October–December 2017) before being delivered to Idaho National Laboratory (INL). At INL, it was stored in an open field shelter for over a year.^14^ A photo of the bales is shown in Figure 1A. Biological heating, the temperature rise caused by the exothermic reactions during the microorganism digestion of the biomass during storage, darkens the color of the biomass. Mildly biologically heated (mild), moderately biologically heated (moderate), and severely biologically heated (severe) materials in the bale were identified visually by their darkening colors (Figure 1B).

**Figure 1:**
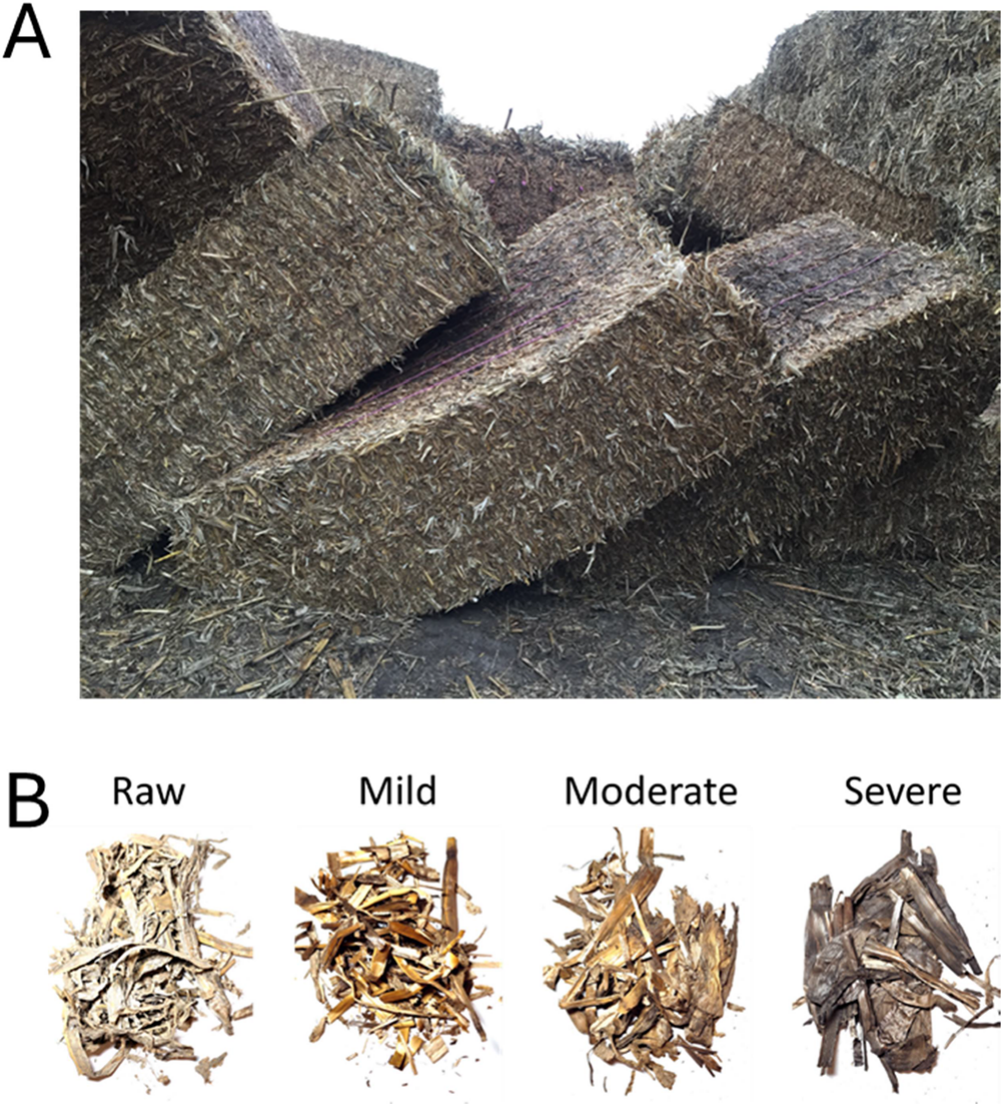
Corn stover material used for this study. (A) Bale of corn stover that was harvested and baled in Iowa. (B) Raw corn stover (raw) showed the lightest color. Elevated biological heating condition caused darker color in the material. The mildly biologically heated (mild), moderately biologically heated (moderate), and severely biologically heated (severe) materials in the bale were identified visually by their darkening colors and then manually sorted.

Fluorescence resonance energy transfer (FRET) have been widely applied to study the subnanometer conformational dynamics down to single-molecule level.^15–17^ Previously, using correlated fluorescence lifetime and stimulated Raman imaging, the reduction of the fluorescence lifetime of lignin in the pretreated biomass was found to be due to the increased lignin chromophore intramolecular interactions in the compact lignin globules.^18^ The increased lignin chromophore intramolecular interactions due to the “collapse” of lignin macromolecular structure were also suggested by in-silica experiments for extracted lignin.^12,13^ The representative fluorescence lifetime decay traces showed faster decay and shorter lifetime for the collapsed lignin (Figure 2A,B). Artificial lignin-carbohydrate complexes were produced by diluting the lignin model compound with carbohydrates in solvent. The fluorescence lifetime from the artificial lignin-carbohydrate complex was elongated as the lignin was in an extended “coil” conformation (Figure 2C).

**Figure 2:**
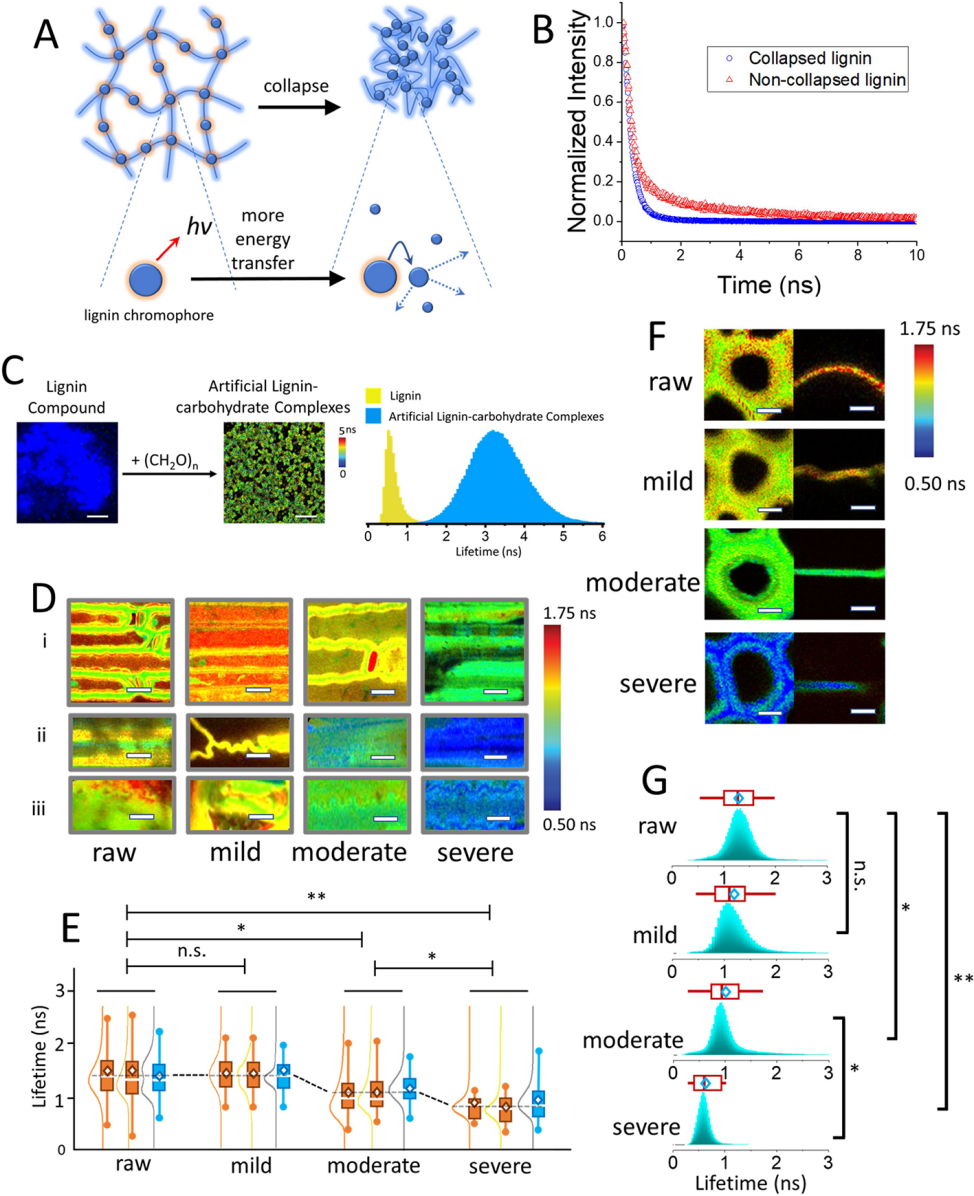
Increased lignin chromophore–chromophore interactions reduced the fluorescence lifetime. (A) Schematic illustration showing the increased fluorescence energy transfer from the chromophore after excitation due to the reduced chromophore–chromophore distance caused by the collapse of lignin. (B) Representative fluorescence decay trajectories showing short and long fluorescence lifetimes from the collapsed and noncollapsed lignin, respectively. (C) FLIM images showing that the short fluorescence lifetime of the isolated lignin can be extended by mixing lignin with carbohydrates, which effectively enlarges the lignin chromophore–chromophore distance and mitigates the fluorescence energy transfer. Fluorescence lifetime histograms show the elongation of lignin fluorescence lifetime by adding carbohydrates. (D) Representative FLIM images of the stalk (i), top (ii), and bottom (iii) of the leaf. (E) Ensemble fluorescence lifetime distributions of samples in panel (D). (F) Representative FLIM images of cell wall cross sections of tracheid cells (left) and parenchyma cells (right). (G) Ensemble fluorescence lifetime distributions that include both the tracheid cells and parenchyma cells. Scale bar in panel (C) 2 μm and panels (D) and (F) 10 μm. Paired Student’s *t* test, *p < 0.05, **p < 0.01, n.s. not significant.

The stalk and leaf fraction of the corn stover feedstock (most of the dry weight of the feedstock) were imaged by fluorescence lifetime imaging microscopy (FLIM). The representative images are shown in Figure 2D. The fluorescence signals from cellulose and hemicellulose/pectin were orders of magnitude weaker than that of lignin, negligible in the presence of lignin.^19–21^ The ensemble fluorescence lifetime histograms constructed from >50 sample pieces are shown in Figure 2E. There was a steady shortening of the lignin fluorescence lifetime correlated with the elevated extent of degradation during storage. Transverse sections of the stalk were prepared to expose the inner cell wall layers for FLIM imaging. The FLIM images of the two major wall types of the materials are shown in Figure 2F. The tracheid cells (Figure 2F, left) contained thick cell walls with a compound middle lamella, cell corner, and secondary wall resolvable under optical microscopy. The lignin fluorescence decay lifetime was slightly shorter at the compound middle lamella and cell corner regions than in other cell locations, consistent with previous observations.^18,22^ The ensemble lifetime distribution histograms from the lifetime distributions of tracheid and parenchyma walls were collected from >50 FLIM images of transverse sections for each cell type. The histograms of lifetime distribution are shown in Figure 2G. There was a consistent lifetime-shortening trend that is similar to the changes on the material surfaces. The reduced fluorescence lifetime suggested more lignin chromophore–chromophore contact, indicating a collapsed form of the lignin network.

Overall, the fluorescence lifetime results provided insights into the subnanometer conformational changes of lignin and suggested that lignin macromolecular structure collapsed into compact globules with more intramolecular contact.

### 2.2 Excitation Modulation Measurements Revealed the Transformation of Lignin into a Globular Form

The plant cell wall comprises highly ordered multiple-layer structures, with cellulose microfibrils showing preferential alignment in the secondary wall’s sublayers. Hemicelluloses and lignin are preferentially oriented alongside cellulose when confined inside the sublayer of orientated cellulose microfibrils.^23,24^ Using lignin’s unique aromatic vibration Raman bands, Agarwal and Ralph discovered that the lignin’s aromatic ring exhibits preferential orientation along the plant cell wall compared to the perpendicular direction to the cell wall surface.^25^

The lignin’s ensemble alignment in the sublayers can be probed by measuring the collective alignment of lignin’s light absorption dipoles using polarized excitation. Herein, we applied the fluorescence modulation depth measurement, a widely adopted microscopic anisotropy tool, to study the collapse of polymer conformations.^26,27^ First, a rotating linearly polarized excitation light excited the sample (Figure 3A and SI). Then, the polarization angle (α) was adjusted by a waveplate to modulate the fluorescence intensity (*I*), following the eq 1:

I(α)∝1+Mcos[2(α−φ)] 1

**Figure 3:**
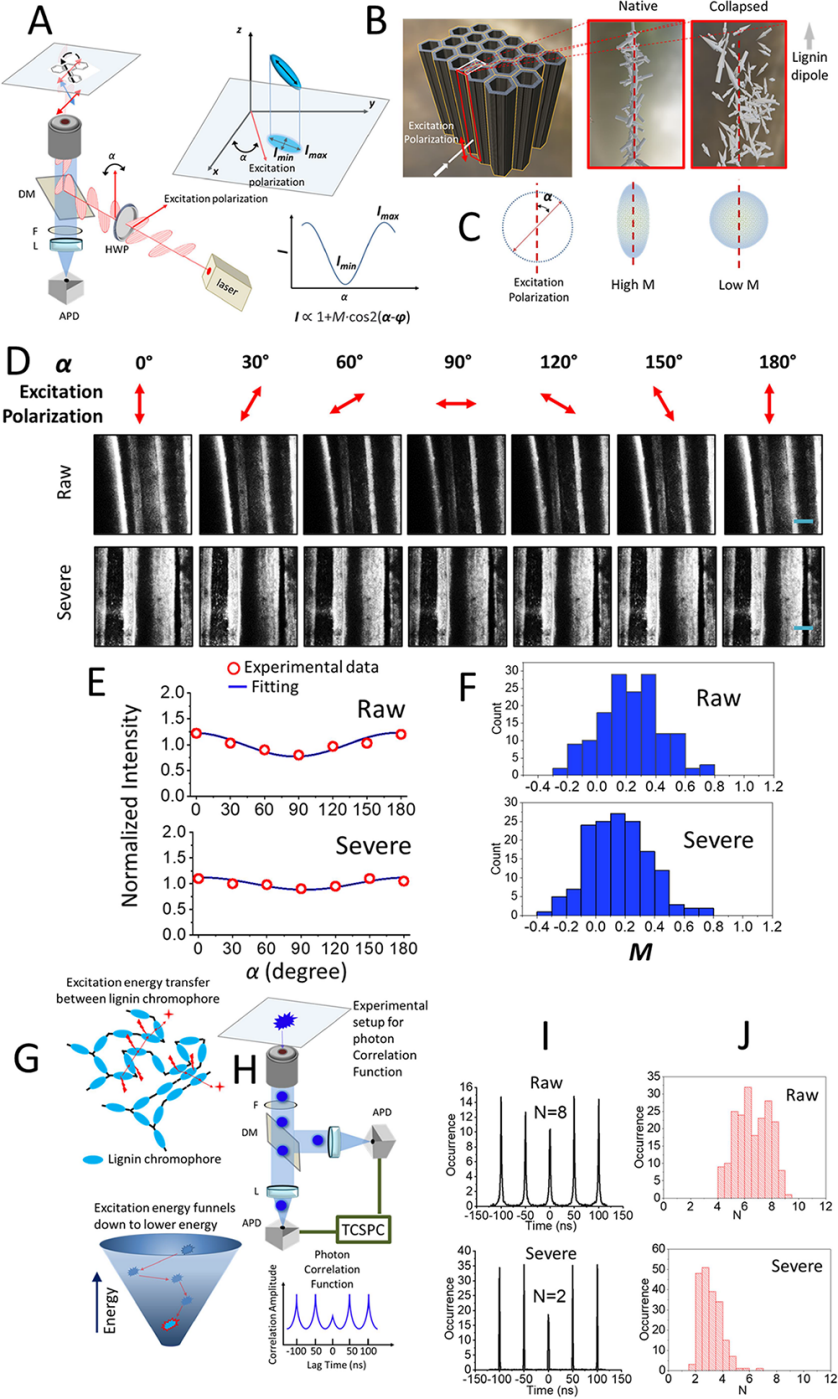
Collapse of lignin probed by polarization microscopy and photon statistic measurement. (A) Fluorescence excitation polarization microscopy was applied to measure the modulation depth of the lignin dipole. (B) Schematic illustration of cell wall bundles concerning the incoming light polarization. The native lignin sandwiched in cell wall sublayers was more ordered than the collapsed lignin. (C) Average diploe alignment of native lignin in the cell wall sublayer exhibits higher modulation depth than the collapsed lignin. (D) Comparison of fluorescence intensity images at varying polarization angle as illustrated in panels (A) and (B). (E) Representative fluorescence trajectories and theoretical fittings to calculate the *M* value. (F) Histogram of M was obtained by fitting a single individual trajectory, as shown in panel (E). (G) Schematic illustration shows the energy transfer from a lignin chromophore in an excited state to a nearby chromophore, eventually funnels down and emits from a smaller number of chromophores at relatively lower energy states. (H) Fluorescence photon antibunching measurement was set up to determine the number of emitters from the photon correlation function. F, filter. DM, dichroic mirror. L, lens. APD, avalanche photodiode. TCSPC, time-correlated single photon counter. HWP, half waveplate. (I) Representative photon correlation functions with the corresponding number of emitters N. (J) Comparison of histograms of the N from the native and collapsed lignin. Scale bar in panel (D), 10 μm.

α is the excitation polarization angle. ϕ is the polarization angle at maximum absorption. *M* is the modulation depth that represents the anisotropy of the absorption/excitation tensor projected on the *x*–*y* plane of the laboratory frame. *M* is related to the morphological order of the individual molecular dipoles. For example, *M* = 1 when the transition dipole moments in a polymer network are all perfectly parallel to one direction, and there is the strongest modulation of fluorescence intensity on the polarization angle. Conversely, *M* = 0 when all the transition dipole moments are random, and *I* shows no modulation by the polarization angle.

Corn stover stalks were imaged with the excitation laser beam perpendicular to the cell wall microfibril bundles (Figure 3B, left). In the natural plant cell wall, lignin molecules were confined along the cellulose microfibrils in the sublayers in the wall (Figure 3B, middle), previously discovered to have orientation preference.^25^ This orientation preference was expected to reduce when there was less spatial confinement (Figure 3B, right). While varying the polarization angle α (Figure 3C, left), the lignin polymers highly confined in a laminate layer (as illustrated in Figure 3B, middle) would exhibit a higher *M* value (as shown in Figure 3C, middle). Conversely, when the lignin network collapsed into a more globule form (as illustrated in Figure 3B, right), a lower *M* value was expected (Figure 3C, right).

The fluorescence intensity *I* versus polarization angle α relationship was extracted from the fluorescence intensity images while the polarization was tuned from 0° to 180°. Figure 3D shows the representative image sequences for the raw and severe samples. The highest fluorescence intensity was observed when the excitation polarization was in the stem direction (along with the cellulose fibril axis). Conversely, the perpendicular polarization alignment produced the lowest fluorescence intensity. The observation of the presential orientation of lignin transition dipole moments on the cell wall was consistent with previous studies.^25^ For each sample region, eq 1 was used to fit the overall fluorescent intensity versus polarization angle and calculate the modulation depth. Representative *I* ∼ α traces and fittings are shown in Figure 3E.

The *M* values obtained for feedstock material without any storage (“raw”) and after the most extensive storage (“severe”) (shown in Figure 3D,E) were 0.23 and 0.12, respectively. The results indicated that (1) lignin showed small orientation preference, and (2) the storage sample exhibited less polarization modulation, which can also be visualized in Figure 3D as the image intensity was less sensitive to polarization angle change. The polarization modulation imaging was performed over many longitudinal section regions for both storage conditions. Then, the same fitting of *M* was performed to build *M* histograms (Figure 3F). From the *M* histogram, in the storage sample, the shift of the polarization anisotropy to a smaller *M* value was apparent. The polarization modulation results suggested the collapse of lignin into a more globular form.

In summary, the reorientation of lignin was detected using the fluorescence excitation modulation depth measurement to assess the anisotropy of the lignin dipoles in the cell wall. The results revealed that lignin polymers in the sublayers of the cell wall collapsed into a globule form after storage.

### 2.3 Photon Correlation Function Analysis Revealed the Reduced Fluorescence Emitters in the Collapsed Lignin

As a result of the increased energy transfer from the excited chromophore to the nearby chromophore (Figure 3G, top), the fluorescence emission was more likely from a smaller number of chromophores that were at the relatively lower energy states in the energy funnel (Figure 3G, bottom). Such fluorescence energy transfer among the multiple chromophores in a polymer network effectively measured the collapse of polymer macromolecular conformation. It can reveal the structural heterogeneity by using single-molecule spectroscopy.^26–28^ Photon correlation function that performed the photon antibunching measurement by splitting the fluorescence signal into two time-correlated single-photon counting detectors (Figure 3H, top and SI). A single fluorescent emitter can only emit one photon at a time. When a 50–50 beam splitter split the fluorescence light into two separated single-photon detectors, only one detector received the photon and produced a counting signal due to the particle nature of light. As the temporal correlation of the counting signals from the two detectors was measured, the coincidence of the counting signal from both detectors shall be zero (at zero-time delay) if only one photon was emitted at a time (Figure 3H, bottom and SI).

Figure 3I shows the representative photon correlation functions from raw and severe samples. The corresponding number of fluorescence emitters, *N*, was calculated using the integrated area of the central peak (i.e., lag time = 0) and the area of the side peaks (SI).^29^ The results revealed a reduced number of emitters after degradation during storage (from *N* = 8 to *N* = 2, shown in Figure 3I). The conclusion was corroborated by the N histograms (Figure 3J) that were constructed by repeating the same photon correlation function measurement over many samples. The reduction in emitters suggested that the lignin structure was collapsed.

Overall, the enhanced intramolecular chain–chain contact due to polymer collapse was discovered as the increased energy transfer to a smaller number of chromophores in a lower energy state. The results suggested that the lignin collapses after carbohydrate degradation during storage.

### 2.4 Fluorescent Aromatic Ring Integrity Was Not Chemically Modified during the Storage

To rule out the possibility that the observed changes in the lignin’s fluorescence could be due to perturbation to lignin’s aromatic fluorescent structure, the storage-induced chemical modifications were investigated by NMR in the aromatic region for lignin as well as in the aliphatic region for carbohydrates. Figure 4A compares aromatic lignin structures before and after storage. There were little changes in the aromatic lignin structures outside of error. The results suggested that the lignin chromophores were not impacted by storage. Significant changes were found in the aliphatic content (Figure 4A) as the loss of hemicellulose signal. Together, the NMR results showed that the loss of hemicellulose was the primary chemical modification during storage. The finding was consistent with a previous study on self-heating, where the microbes digested the accessible carbohydrates in the feedstock.^30^

**Figure 4:**
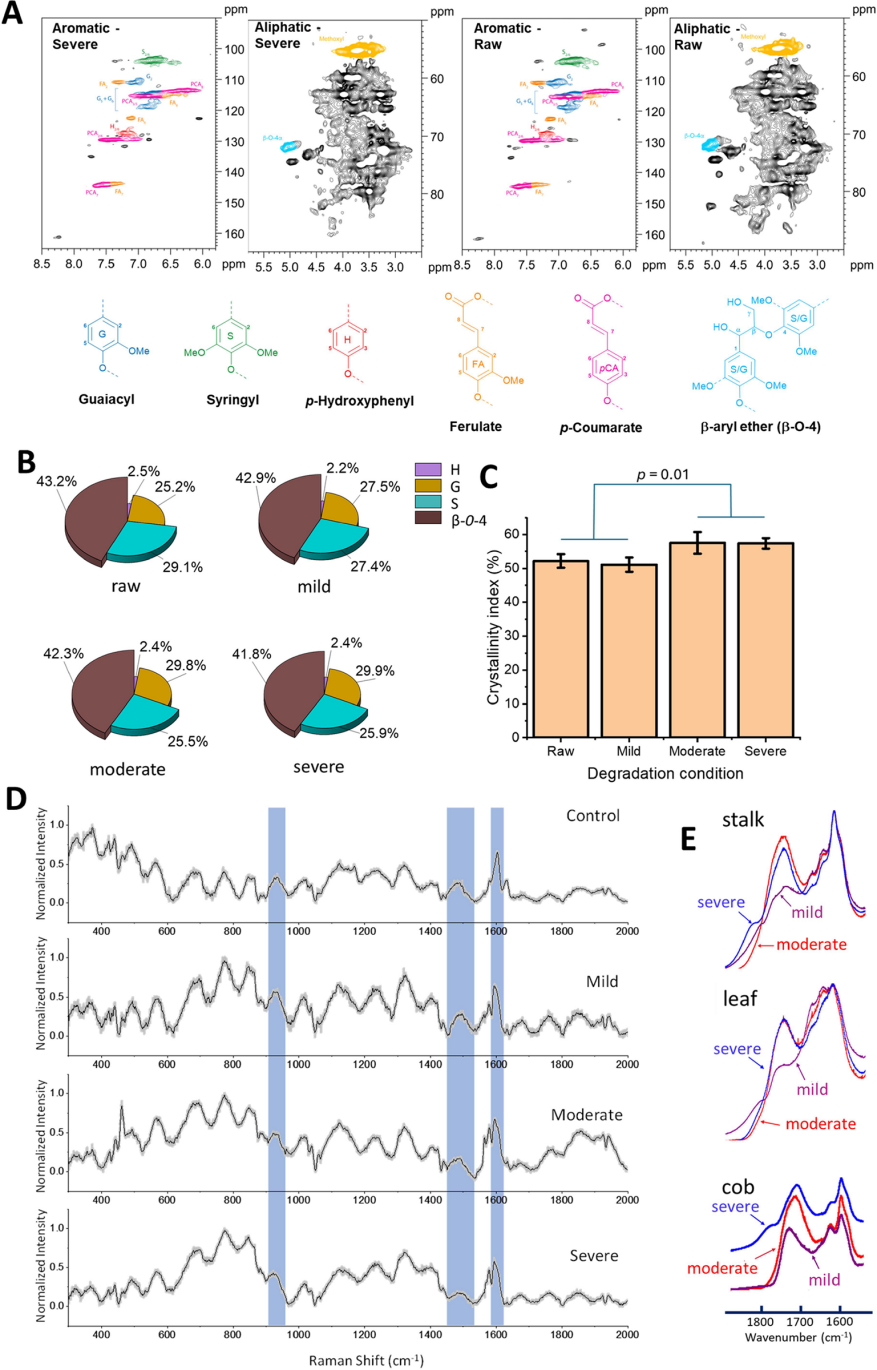
Carbohydrates particularly hemicellulose were impacted by self-heating while chemical modification to lignin structure was limited. (A) NMR revealed more changes in the aliphatic than aromatic region. (B) Comparison of the S, H, and G lignin content and β-O-4 content. (C) Cellulose crystallinity measured by XRD. There was a significant increase in crystallinity index for the group of moderate and severe verse the group of raw and mild degradation conditions. *p* value was determined by using one way ANOVA analysis for the four groups. (D) Raman spectra of the stalk fraction of the feedstock. (E) IR spectra of stalk, leaf, and cob fractions of the feedstock.

The quantification of S, H, and G lignins remained unchanged following elevated storage conditions (Figure 4B), supporting the conclusion that the lignin chemical structure received minimal microbial digestion. Comparisons of the S, H, G, ferulate, and coumarate content before/after storage showed little changes in their content (Figure S4).

In summary, the NMR results show minimal chemical modification to the lignin aromatic ring structure. Therefore, the fluorescence changes observed in this study were not from the modification of lignin chromophores.

### 2.5 Reorganization of the Wall Polymer Microenvironment Was Confirmed by the Changes in Cellulose Crystallinity

We hypothesized that removing hemicellulose in the wall polymer matrix lifted the spatial confinement and allowed for the space necessary to reorganize wall polymers. The improved crystallinity of cellulose observed such reorganization of the wall polymer microenvironment once the hemicellulose is removed. Powder X-ray diffraction measurements showed an increase in cellulose crystallinity index (CrI) after storage (Figure 4C), representative raw X-ray spectra are shown in Supporting Information (Figure S5). In addition, removing hemicellulose created room for the cellulose microfibers to realign and form a more ordered crystalline structure. Thus, the loss of hemicellulose due to microbial digestion during storage would increase cellulose crystallinity, which was consistent with previous works that found the increase of cellulose crystallinity was often associated with reduced hemicellulose concentrations.^31^

### 2.6 Raman Results Showed a Steady Reduction in Xylan and No Change in the Lignin Aromatic Ring

The lignin’s Raman band at 1600 cm^–1^, associated with the lignin’s aromatic ring, showed no change (Figure 4D). Detailed analysis and comparison are shown in Supporting Information (Figure S1 and Table S1). The degradation of the hemicellulose, such as xylan, was observed in the Raman spectra by the reduced xylan-associated peaks at 920 and 1470 cm^–1^ (Figure 4D). Detailed analysis and comparison are shown in Supporting Information (Figures S2, S3, Tables S2, and S3). The 920 cm^–1^ peaks were the C–O–C stretching coupled with C=C ring stretching in xylan.^32^ The 1470 cm^–1^ peak was associated with xylan, O–H, and CH2 vibrations^33^ and was previously used for the label-free stimulated Raman imaging of the xylan content in corn stover during enzymatic digestions.^32^

### 2.7 Conservation of Lignin Aromatic Structure Was Confirmed by IR

Two infrared spectroscopic techniques, ATR (attenuated total reflectance) and DRIFTS (diffuse reflectance infrared Fourier transform spectroscopy), were applied to investigate the IR region 1650–1800 cm^–1^ (Figure 4E). The 1620–1640 cm^–1^ region was associated with C–O conjugation in quinones coupled with C=O stretching. Only minor changes were observed in this region. The 1600 cm^–1^ region was associated with the aromatic C=C skeletal vibrations of the benzene ring in lignin. Little change in absorbance intensity was observed at 1600 cm^–1^ for all anatomical fractions and degrees of degradation. The absence of any significant changes in absorbance intensity was consistent with lignin collapse and coalescence while preserving the aromatic nature of lignin, which was also consistent with the Raman results for preserving the aromatic ring nature. The 1700–1750 cm^–1^ peak was the C=O stretching in unconjugated groups (or alternating double bonds), reflecting changes in C=O functional groups (e.g., carbonyls, esters, ketones, aldehydes, and carboxylic acids) in hemicelluloses. An increase in the degree of biological degradation resulted in an increase in the absorbance in this region. Shifts in the peak wavenumbers were likely attributed to changes in the local chemical environment due to the conjugation of carbonyl groups to additional C=O bonds from thermal oxidation or thermal degradation. Overall, these results from IR provided insights into the chemical changes occurring during the degradation of hemicellulose and preservation of lignin in the wall polymer matrix.

### 2.8 Collapse of Lignin Negatively Affected the Monomer Yield from Lignin-First Alkaline Oxidation

Lignin valorization approaches, such as depolymerizing lignin via alkaline oxidation, are critical to biorefining to produce phenolic aldehydes, acids, ketones, and aliphatic acids as high-value products. Sr­(OH)_2_ in replacement of NaOH can be more beneficial in terms of process economics.^34^ However, the efficiency of this process can be affected by various factors. The impact of feedstock storage, particularly the collapse of lignin structure on this alkaline oxidation using Sr­(OH)_2_, was unknown. Our results showed a significant drop in total lignin monomer yield, indicating that lignin collapse negatively affected the efficiency of the alkaline oxidation (Figure 5A).

**Figure 5:**
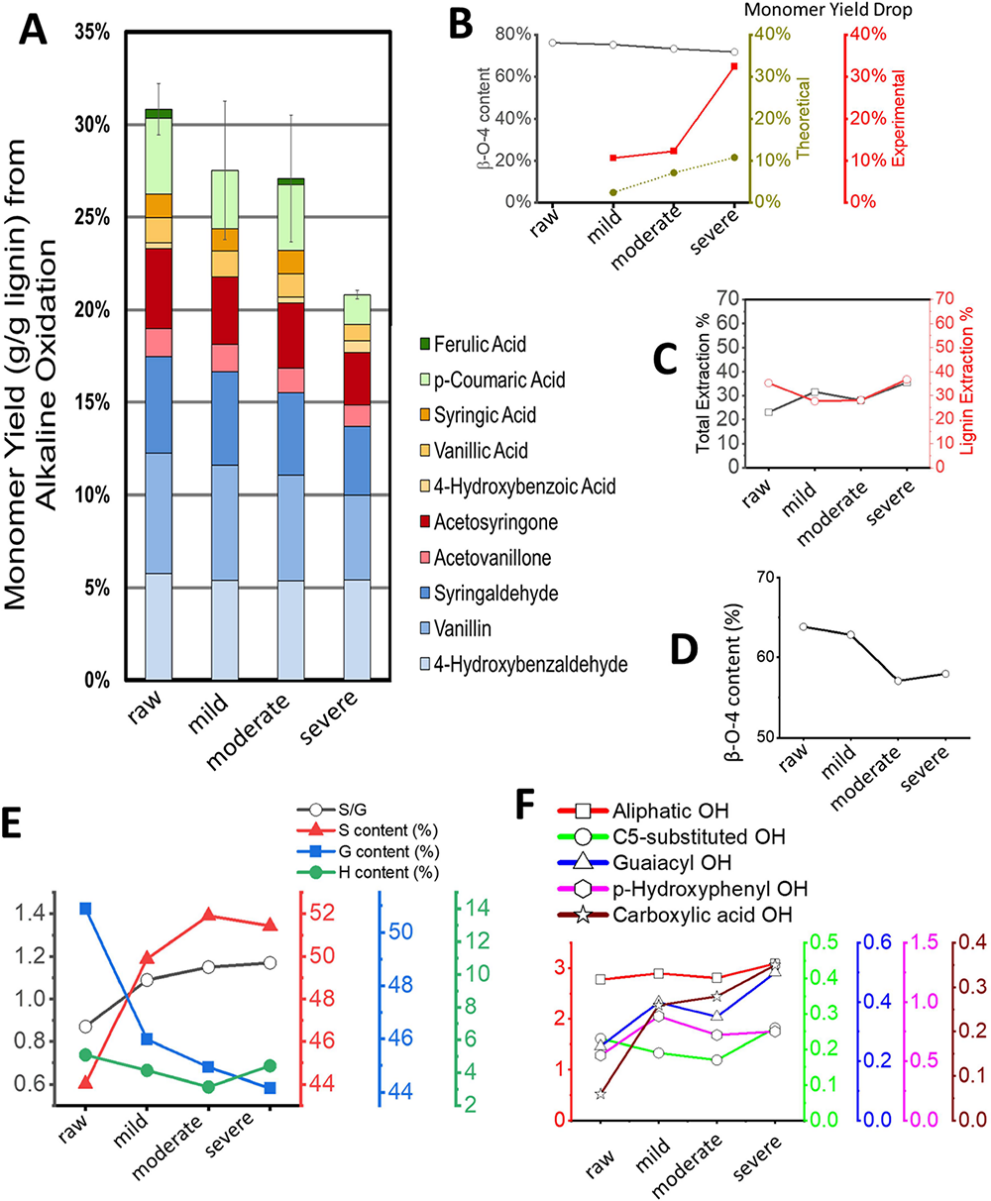
Impacts on lignin depolymerization and extracted milled wood lignin. (A) Monomer yields from alkaline oxidation. (B) Total alkaline oxidation monomer yield drop exceeded the theoretical estimate based on the β-O-4 content. (C) Total mass extraction yield and the lignin extraction yield during the milled wood lignin extraction were not significantly impacted. (D) β-O-4 content in the extracted milled wood lignin was reduced. (E) Comparison of S, G, and H content in the milled wood lignin. (F) Comparison of the hydroxyl groups in the milled wood lignin. The unit of the measurements was mmol/g.

Lignin monomer yield from alkaline oxidation was sensitive to the β-O-4 content as cleavage β-O-4 generates monomers. The theoretical maximum monomer yield can be empirically estimated as the square of the abundance of the β-O-4. However, our results showed that there were minimal changes in the β-O-4 content induced by storage, and the experimental drop in lignin monomer yield was far more significant than the theoretical estimate (Figure 5B). The lower-than-theoretical monomer yields suggested that the collapse of the lignin may inhibit depolymerization by limiting the access to hydroxide ions or active oxygen to the lignin bonds. Since using Sr­(OH)_2_ in replacement of NaOH can be more cost-effective to valorize lignin, the results highlighted the importance of considering the collapse of lignin structure in designing alkaline oxidation.

### 2.9 Collapse of Lignin Affected the Extracted Wood-Milled Lignin

The lignin extraction following the standard wood lignin extraction procedure was also applied to investigate the impact of lignin collapse. In contrast to the significant changes in solubility of cellulose and hemicellulose, the overall lignin extraction yields were slightly reduced (<8%) after mild and moderated storage and only slightly lifted (∼2%) after severe storage (Figure 5C). After storage, the β-O-4 ether linkages were up to 8.8% less in the extracted lignin (Figure 5D). The collapse of lignin impacted the structure of the extracted lignin as a steady increase in the S/G ratio (Figure 5E).

Higher aliphatic hydroxyl, corresponding to β-O-4 ether cleavage, was also observed (Figure 5F). The increasing concentration of carboxylic acid hydroxyl in the extracted lignin supported the increased intensity of C=O in the IR spectrum. In addition, with the cleavage of β-O-4 ether, lignin condensation was observed in NMR (Figure S7). The results suggested that the collapsed lignin was more easily condensed during a pretreatment process, which was critical to the quality attributes and product yields of the lignin conversion process.

In summary, biological self-heating exhibited broad impacts on the structural properties of the extracted lignin. These attributes included cleavage of β-O-4 ether linkages, changes in S/G units, and increased carboxylic acid hydroxyl groups, which provided information to understand the biological self-heating mechanism and underlying lignin conversion processes.

## 3 Discussion

In this study, we utilized multiple fluorescence microscopic techniques in combination with other spectroscopic methods to characterize the corn stover feedstock after storage. We discovered the collapse of lignin macro-molecule conformation induced by storage. We investigated the impact of lignin collapse on lignin monomer extract and milled wood lignin extraction, two standard methods for lignin valorization in the lignin-first scheme. In the lignin monomer extraction process, the collapsed lignin led to lower-than-theoretical yields and monomer production, due to the inhibited depolymerization from the limited access of hydroxide ions or active oxygen to the lignin bonds. In the milled wood lignin extraction, the collapse of lignin in the feedstock altered the structural properties of the extracted lignin, including the cleavage of β-O-4 ether linkages, changes in S/G units, and increased carboxylic acid hydroxyl groups extracted lignin.

While hemicellulose degradation is central to lignin collapse, microbial activity during storage may synergistically exacerbate this process. For instance, pectin—a key component of the plant cell wall matrix—is susceptible to enzymatic cleavage by microbial communities (e.g., *Aspergillus* or *Bacillus* spp.).^35^ Pectin degradation could further destabilize the cell wall architecture, reducing the polysaccharide matrix that supports lignin’s spatial distribution.^36^ Additionally, certain microbes produce lignin-modifying enzymes (e.g., laccases or peroxidases), though their role in storage-induced lignin restructuring remains speculative. Future studies profiling microbial consortia and their enzymatic activity during storage could clarify these interactions. Environmental factors such as elevated moisture levels may act as a double-edged sword: (i) facilitating hydrolytic hemicellulose breakdown (even abiotically)^37^ and (ii) promoting microbial proliferation.^38^ Water can also plasticize lignocellulosic structures, potentially accelerating physical reorganization.^39^ Conversely, repeated wet–dry cycles might induce mechanical stress, compacting biomass and altering lignin accessibility.^40^ Other environmental factors such as higher temperatures could accelerate both enzymatic/abiotic hemicellulose hydrolysis and microbial metabolism.^41^ Thermal fluctuations may also drive lignin mobility, enabling coalescence into less reactive aggregates.^42^ Controlled experiments isolating temperature’s effect on lignin’s glass transition temperature could resolve this. The confluence of hemicellulose loss, pectin degradation, and environmental stressors likely creates additive or synergistic effects on lignin collapse. For example, moisture-driven microbial activity might amplify hemicellulose and pectin breakdown, while temperature modulates reaction rates. Such interactions could yield lignin with distinct physicochemical properties (e.g., hydrophobicity, porosity), directly impacting valorization efficiency.^43^ Under the same mechanical milling conditions, severely baled feedstock produced more finer particles than less baled feedstock counterpart (Figure S6 and Table S4). For instance, collapsed lignin may exhibit reduced enzymatic digestibility or altered reactivity in catalytic depolymerization.

## 4 Materials and Methods

A concise summary of the experimental methods is shown in this section. Detailed descriptions of the materials and techniques are in Supporting Information. FLIM, fluorescence polarization measurements, and photon antibunching statistics were performed at National Renewable Energy Laboratory on a custom fluorescence microscope based on the PicoQuant MT200 system attached to an Olympus IX 81 microscope. NMR 2D HSQC and ^31^P NMR measurements were performed at National Renewable Energy Laboratory and Idaho National Laboratory. X-ray powder diffraction measure of crystallinity index was performed at Lawrence Berkeley National Laboratory. ATR and DRIFTS were performed at Idaho National Laboratory, Los Alamos National Laboratory, and Sandia National Laboratory. Lignin oxidative depolymerization was performed at National Renewable Energy Laboratory according to the previous procedure.^34^

## Supplementary Material



## Notes

Additional information on sample materials; detailed description of instrument setups, sample measurement methods and data analysis procedures for fluorescence microscopy, Raman spectroscopy, NMR, IR, and X-ray diffraction; experimental procedures for the alkali oxidation of lignin in the feedstock and the milled wood lignin extraction; and particle size analysis results from the mechanical fractionation of the feedstock materials after different storage conditions (PDF)
