# Supplementary material for: Storage-Induced Collapse of Lignin Macromolecular Structure and Its Impacts on the Biorefinery
Source: ACS Sustain Chem Eng. 2025 Jul 18;13(30):12178–87. doi: 10.1021/acssuschemeng.5c04284 (PMC12326388; doi:10.1021/acssuschemeng.5c04284)
Supplement: Supplementary file 1 [file sc5c04284_si_001.pdf]

Supplementary document for:

Storage-induced collapse of lignin macromolecular structure and its impacts on the biorefinery

Yining Zeng[1], Kuan-Ting Lin[2], Renee M. Happs[1], Juan H. Leal[3], Xihui Kang[4], Chang Dou[4], Jacob S. Kruger[1], Ling Ding[2], Kenneth L. Sale[5], Troy A. Semelsberger[3], Allison E. Ray[6], Ning Sun[4] and Bryon S. Donohoe[7*]*

1-Renewable Resources and Enabling Sciences Center, National Renewable Energy Laboratory, Golden, CO 80401, USA.

2-Energy and Environmental Science and Technology, Idaho National Laboratory, Idaho Falls, ID 83415, USA.

3-Material Physics Applications Division, Los Alamos National Laboratory, Los Alamos, NM 87545, USA.

4-Advanced Biofuels and Bioproducts Process Development Unit, Biological Systems and Engineering Division, Lawrence Berkeley National Laboratory, CA 94608, USA.

5-Computational Biology and Biophysics, Sandia National Laboratories, Livermore, CA 94500, USA.

6-Science and Technology, Idaho National Laboratory, Idaho Falls, ID 83415, USA.

7-Biosciences Center, National Renewable Energy Laboratory, Golden, CO 80401, USA.

*To whom correspondence may be addressed: Yining.Zeng@nrel.gov and Bryon.Donohoe@nrel.gov.

Supporting Information Summary

Supporting Materials and Methods	pg S2–S8
Figure S1	pg S9
Figure S2	pg S10
Figure S3	pg S11
Figure S4	pg S12
Figure S5	pg S13
Figure S6	pg S14
Figure S7	pg S15
Table S1	pg S16
Table S2	pg S17
Table S3	pg S18
Table S4	pg S19
Supporting References	pg S20

Supporting Information Materials and Methods

Biologically degraded corn stover samples

Corn stover was harvested and baled in Story County, Iowa, between October 12 and 27, 2017[1]. Biological heating, the temperature rise caused by the exothermic reactions during the microorganism digestion of the biomass during storage, darkens the color of the biomass [2]. Mildly biologically heated (mild), moderately biologically heated (moderate), and severely biologically heated (severe) materials in the bale were identified visually by their darkening color.

The severe samples were collected from bales that had the darkest color. Over half of the bale length was severely self-heated. The darker material was collected from the bale as it was flaked apart in 3 places. In each location, approximately 6 samples were taken across the flake face (18 samples total). All samples were combined.

A portion of the bale was moderately self-heated, identified as a color transition between severely self-heated (the darkest) and not self-heated (the lightest color). The bale was flaked apart at 2 locations, and approximately 6 samples were collected from across each flake on either side of the split bale to generate 12 samples. All samples were combined.

The mildly degraded materials are the portion of the bale that showed minimal signs of self-heating that were flaked apart at two locations, and 3 to 6 samples were collected from across each flake for ~9 grab samples total. All samples were combined.

Fluorescence lifetime imaging microscopy (FLIM)

Fluorescence lifetime imaging was performed on a Picoquant MicroTime 200 system attached to an Olympus IX71 inverted microscope base. The Olympus microscope had a high numerical aperture water immersion objective lens (UPlSApo 60X 1.20 NA W, Olympus). The PicoQuant MicroTime 200 was equipped with a 405 nm excitation laser operating up to 40 MHz (PicoQuant LDH-P-C-405B). The sample was placed on a raster-scanning stage (Physik Instrumente), and the epi-fluorescence signal was collected, filtered by a 430 nm long-pass filter (Chroma HQ430LP), and detected by a single-photon avalanche diode (Micro Photon Devices). The TTL signal from the single-photon avalanche diode was sent to the Picoquant PicoHarp 300 for time-correlated single photon counting (TCSPC) through the Time-Tagged Time-Resolved (TTTR) data acquisition T3 mode. The FLIM image was captured with 512 x 512 pixels resolution at 0.8 ms per pixel dwell time. Laser intensity was kept at ~ 0.01 μW at the sample to minimize photobleaching damage while obtaining an adequate signal-to-noise ratio. FLIM images and lifetime analysis were performed by PicoQuant SynPhoTime (V5.3.2.2). For our corn stover materials, when the excitation power was lowered to avoid photodamage to the sample, there was still a strong fluorescence signal. The signal intensity level is consistent with the lignin fluorescence in our previous studies using lignin model compounds[3]. However, due to the complex nature of lignin polymers, determining the exact number of decay components is challenging. Thus, to obtain the fluorescence decay lifetime, we adopt the average decay lifetime from fitting the fluorescence decay trace measured by PicoQuant SynPhoTime.

Fluorescence excitation polarization measurement

The fluorescence excitation polarization measurement was performed on the same apparatus as FLIM with modifications. A half-waveplate was added to the excitation laser beam path to allow the manual rotation of the polarization. A second polarizer was used to check the linearity of the polarization after the objective lens. The polarization extinction was > 1:200 after the objective lens. The excitation laser power fluctuation after the objective lens during the polarization rotation was <1%. Laser intensity was kept at ~ 0.01 μW at the sample to minimize photobleaching damage and maintained during polarization rotation. The sample focus on the Z-axis was monitored by imaging the back-reflection spot from the sample on a CCD video camera. Before each image acquisition, the sample focus was maintained at the same position to avoid any image intensity fluctuation due to the drift in sample focus. The fluorescence intensity image was acquired through the Time-Tagged Time-Resolved (TTTR) data acquisition T3 mode, while only the intensity was used in producing the image. The stalk anatomic fractions of similar size were hand-picked for those samples of different baling severities. A longitudinal section was cut from the stalk for microscopic observation. The sample section was sandwiched between two coverslips and taped on the microscope stage to avoid intensity variation due to sample drift.

Photon correlation function

The photon correlation function measurement was performed on the Hanbury Brown-Twiss interferometer set-up scheme for probing photon antibunching. The Olympus microscope and PicoQuant MT200 were modified by add-in a 50-50 beam splitter to split the epi-fluorescence signal, and two single-photon avalanche diodes were set up to collect the split fluorescence signal. The signals from the two single-photon avalanche diodes were acquired by PicoHarp 300 through the PicoQuant T2 mode. The following modifications in the cable connection series were made to convert from the T3 mode used in imaging mode to the photon correlation T2 mode: 1) an ATM10 attenuator was added into each signal cable from the single-photon avalanche diode; 2) disconnected the PHR800 Router control cable from the PicoHarp PH300 controller; 3) connected the two attenuated signal cables to PicoHarp PH300 Channel 0 and 1 ports, respectively. For T2 mode, discriminator settings for the two channels were adjusted to 150 mV for both channels in the SymPhoTime Settings. The sample was excited by the 405 nm laser excitation at 20 MHz. The excitation power was kept at ~ 0.01 μW after the objective lens to minimize photobleaching. The duration of each data acquisition was limited to 15 sec to avoid artifacts due to photodamage. The

photon correlation peaks at 0 and 50 ns were integrated to calculate the number of emitters in the focal volume. The number of emitters, N, was calculated from $1/N = 1 - (A_c/A_s)$, where A_c is the area of the central peak showing at time 0, and A_s is the area of the side peak at time 50 ns.

Raman spectroscopy

Raman spectra were acquired on a LabRam HR800 confocal Raman system (Horiba Jobin Yvon, Edison, New Jersey, United States) attached to an Olympus BX41 microscope. To minimize the impact of autofluorescence from biomass, a 785 nm diode laser source was used. Samples were placed on a glass cover slide, and the probing laser beam was focused above the sample through a 40X objective lens (Olympus, UPlanSApo, 0.95 NA). The same objective lens collected the Raman signal from the sample. Integration time for spectrum acquisition was varied so that the maximum Raman signal intensity is about ~80% of the full dynamic range of the CCD detector. More than ×30 accumulations were acquired and averaged for each spectrum to enhance the signal-to-noise ratio. For each self-healing level, >20 sample locations among sample particles were measured to include the variation of sample heterogeneity. Each spectrum was first normalized and then the average and standard deviation of all the spectra from the same condition were calculated.

To compare Raman band intensity, the local minima were used to identify the Raman band of interest. For example, the 920 cm^{-1} band for hemicellulose was identified from 903 to 967 cm^{-1}. Its band intensity was integrated from the above frequency range, and with its baseline background intensity integrated and subtracted. Using the same method, 1470 cm^{-1} band was integrated in the range from 1446 to 1540 cm^{-1}, and 1600 cm^{-1} band was integrated in the range from 1582 to 1617 cm^{-1}. The Y-axis has a normalized, unitless scale. Therefore the unit of the peak area is the same cm^{-1} as X-axis.

Nuclear magnetic resonance (NMR) spectroscopy

For ^{31}P detection, all the lignin solutions were transferred into a 5-mm NMR tube via a glass Pasteur pipette. Then, ^{31}P NMR analysis was conducted with a Bruker Avance III HD 500 MHz spectrometer. First, an inverse gated decoupling pulse was used to acquire the ^{31}P spectra with a spectral width (SW) of 100 ppm, the center spectrum was 140 ppm, and the acquisition time was 0.8s. As a result, 64 or more scans were acquired at a relation delay greater than or equal to

10s. Next, NMR processing commands were applied to process the free induction decay signal to the spectrum and to perform automatic phase correction, and finally, different hydroxyl groups were calculated.

For heteronuclear single quantum coherence (HSQC), the biomass samples were ball milled, and 30–50 mg of the solid was then dissolved in 500 μL DMSO-d6 and pyridine-d5 (4:1, 500 μL). HSQC NMR spectra were acquired at 40 °C on a Bruker Avance III 600 MHz spectrometer at 11.7 T using a room-temperature broadband probe. Spectra were acquired across 1024 points and an SW of 12 ppm in the F2 (1H) dimension and 256 points and SW of 220 ppm in the F1 (^{13}C) dimension. The spectral processing and integrations were performed using TopSpin 3.6. Fig. S4 shows that ferulate and coumarate only exhibit small changes while S, G, and H content remain.

X-ray diffraction (XRD)

Advanced Light Source (ALS) beamline 12.2.2 at Lawrence Berkeley national laboratory was used for powder XRD measurement. Each biomass sample was cryo-milled (SPEX 6875D, SPEX Sample Prep, Metuchen, NJ) for five minutes in liquid nitrogen to prevent any possible thermal degradation from the heat generated from the milling. Samples were placed in plastic capillaries and attached to magnetic goniometer bases (MiTeGen, MicroRT, Ithaca, NY). An X-ray beam at 40 KeV and 20 mA with a polarization factor of 0.99 illuminated the samples. Each sample's X-ray diffraction of an empty capillary was measured as a baseline scan to subtract the background contribution. Scans were conducted between 2θ in the range of 3° - 40°. Samples were moved along their y-axis during each scan to maximize the sampling area in the incident X-ray beam. A Mar-345 image plate (marXperts GmbH, Norderstedt, Germany) detector was used to gather diffraction data. Crystallinity index values were determined by the Segal method[4, 5]. The CrI of corn stover was calculated as per the well-established equation below[6, 7]:

$$\text{CrI} (\%) = (I_{002} - I_{am})/I_{002} \times 100\%$$

where I_{002} is the maximum peak intensity at 2θ of 22.5°, while I_{am} is the intensity diffraction of amorphous cellulose at 2θ of 18.4°.

The raw X-ray data with one representative run for each sample condition are shown in Fig. S5

Infrared spectroscopy (IR)

Two different infrared spectroscopy techniques were used to probe the changes in cellulose, hemicellulose, and lignin composition for the cob, leaf, and stalk anatomical fractions for the mild and severe cases of biological degradation. The two FTIR techniques were ATR (attenuated total reflectance) and DRIFTS (diffuse reflectance infrared Fourier transform spectroscopy). Model cellulose, hemicellulose, methylcellulose, hydrolytic lignin, and organosolv lignin were used to collect baseline spectra.

Alkali oxidation of lignin in the feedstock

Lignin alkali oxidation was carried out in a 75 mL Parr reactor. Solid biomass 0.3 g and 30 mL 1 M $Sr(OH)_2$ in deionized water were added to the Parr reactor and purged with He gas after the reactor was sealed. The He pressure was adjusted after the leaks were checked. Then the reactor was heated to 175 °C. The pressure inside the reactor was raised to roughly 8 bar before adding 22 bar partial pressure of air to reach 30 bar total pressure. After 10 min oxidation time, the reactor was quenched in room temperature water.

Milled wood lignin extraction

Baled corn stover feedstock was first ball-milled before lignin was extracted by 0.05M HCl in 80% dioxane aqueous solution at 85°C for 4 hours. The extracted lignin was precipitated by evaporating dioxane and adding water. Then the lignin solid was washed with water and freeze-dried to collect the final milled wood lignin. Cellulose and hemicellulose are also partially extracted from biomass during the same process.

Mechanical fractionation of feedstock and particle size analysis

Feedstocks were mechanically fractionated using the same procedures. All samples were separated according to the severity of baling and then processed through three stages of milling. First, samples were passed through a Vermeer BG480 bale processer fitted with a 75 mm screen. Second, a Bliss Hammermill with a 25 mm screen was used to further mill the samples. Third, the

fine particles from the previous hammermill were further milled using a Thomas Model 4 Wiley knife mill (Thomas Scientific, Swedesboro, NJ) fitted with a 2 mm screen. Total 21 samples (Table S4) were produced and placed in separate plastic grab bags (25 g). Particle size analysis of the feedstock fragments was performed on a Camsizer 3D particle size and shape analyzer (Microtrac MRB, USA). The particle size distribution measured as Feret length ranging from 20 μm to 30 mm was analyzed. The histograms were first normalized by volume and then fit by a log normal distribution function previously established to describe particle size distributions [8]:

$$y = y_0 + \frac{A}{wx\sqrt{2\pi}} e^{\frac{-[ln\frac{x}{xc}]^2}{2w^2}}$$

> 650000 particles of each type of material were analyzed to include large set of variability and better represent the sample size distribution. Both the histograms and fittings (Fig. S6) clearly demonstrated that feedstock after storage yielded a higher fraction of smaller fragments. This could be due to an increased brittleness of the material and/or other changes in the mechanical properties. Further investigation on the structure-mechanical relationship is currently ongoing.

Supporting Information Figures and Tables

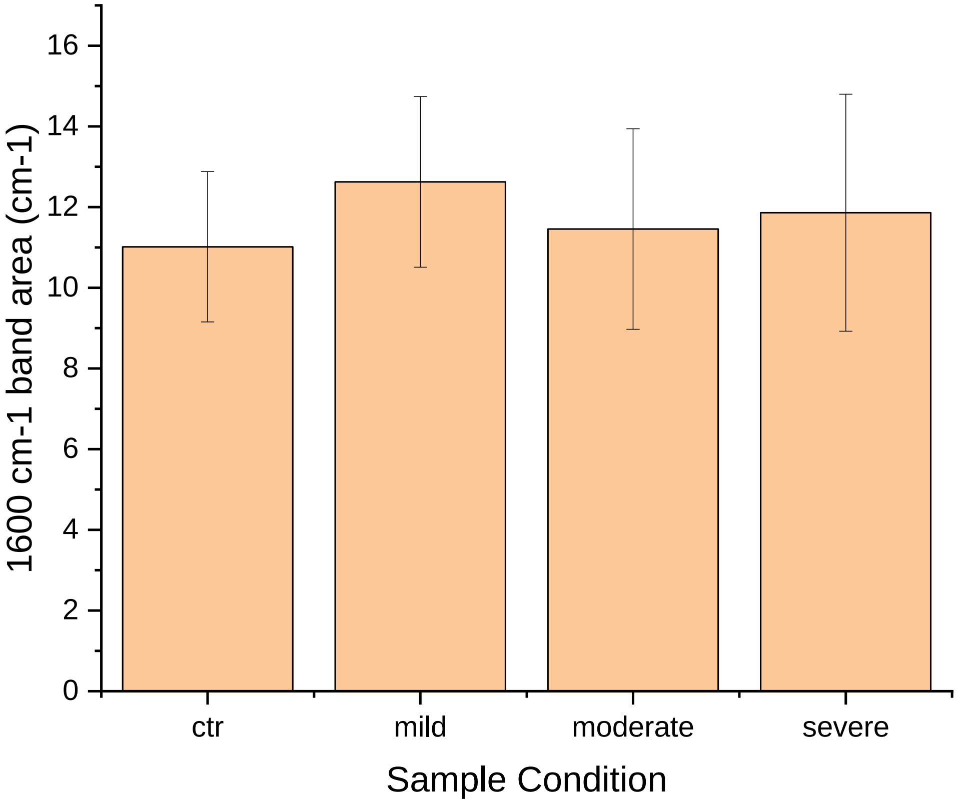

Figure S1. Comparison of the 1600 cm^{-1} lignin aromatic Raman band intensity.

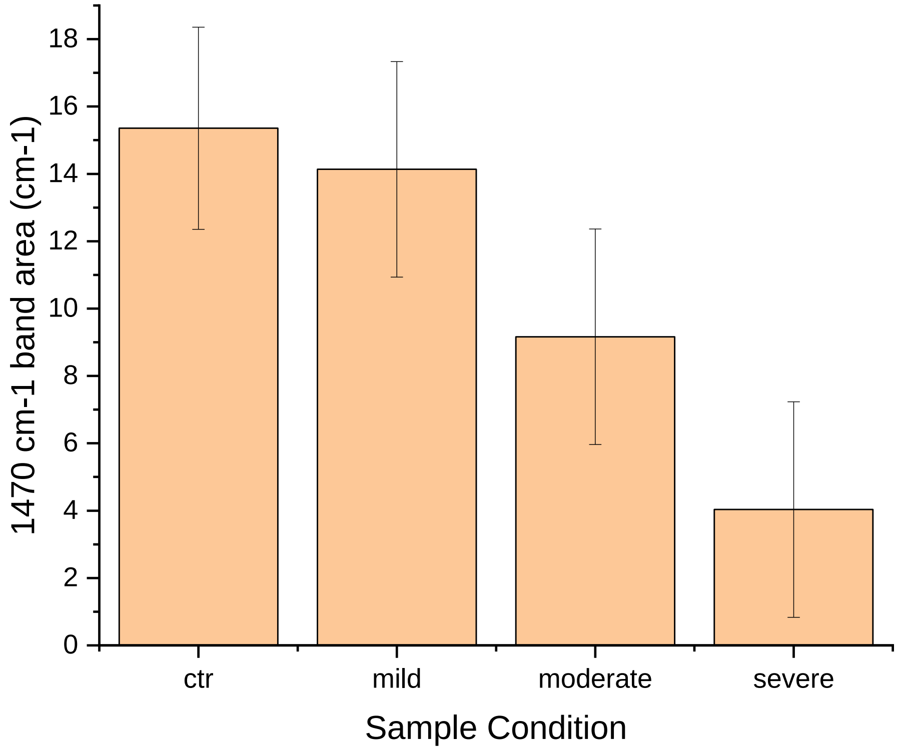

Figure S2. Comparison of the 1470 cm^{-1} xylan Raman band intensity.

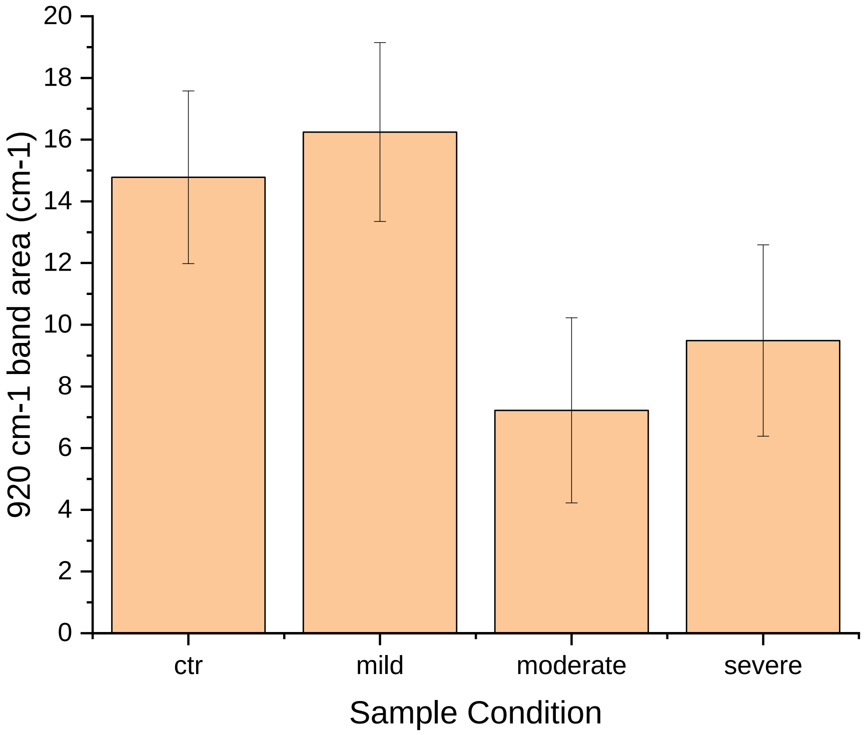

Figure S3. Comparison of the 920 cm^{-1} xylan Raman band intensity.

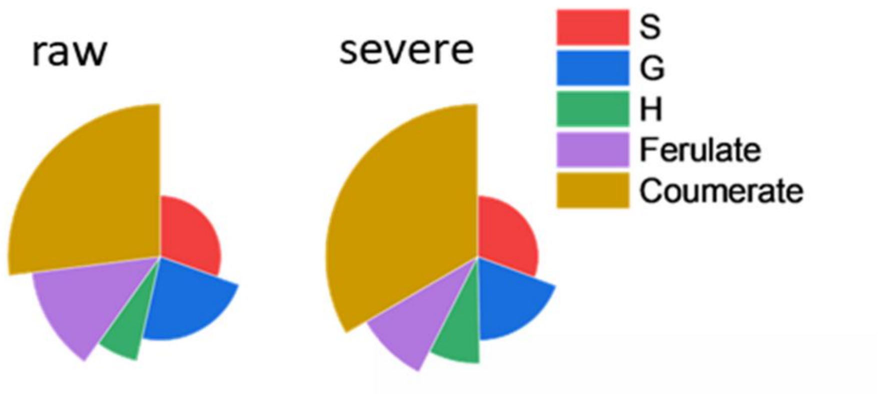

Figure S4. Comparison of the ferulate, coumarate content together with the S, G, and H type of lignin content (%) in raw and severe samples.

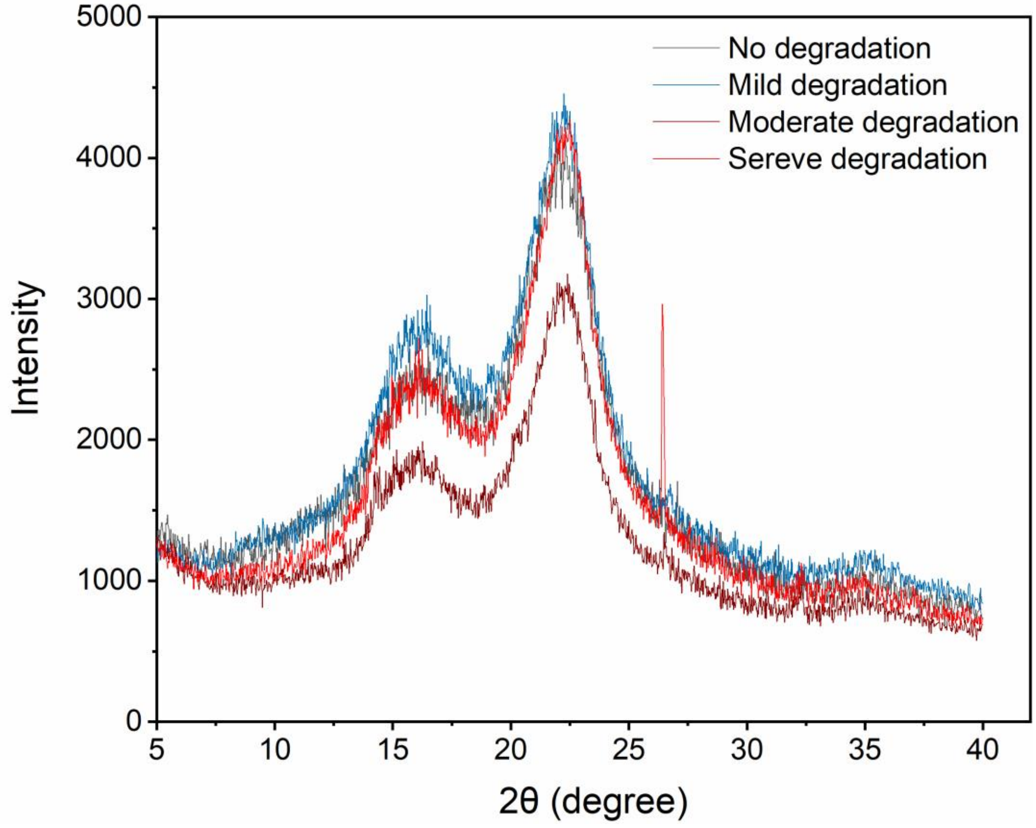

Figure S5. The raw X-ray data that shows one representative spectroscopic run for each sample condition.

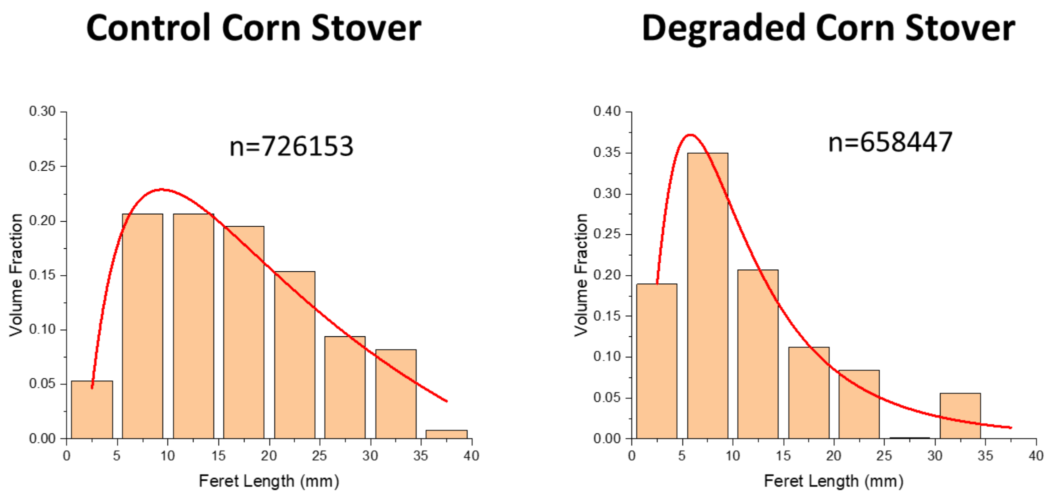

Figure S6. Comparison of particle size distributions of the feedstock fragment after mechanical fractionation. Histograms are fitted with a log normal distribution function (red). The number of fragments is shown in the corresponding panel.

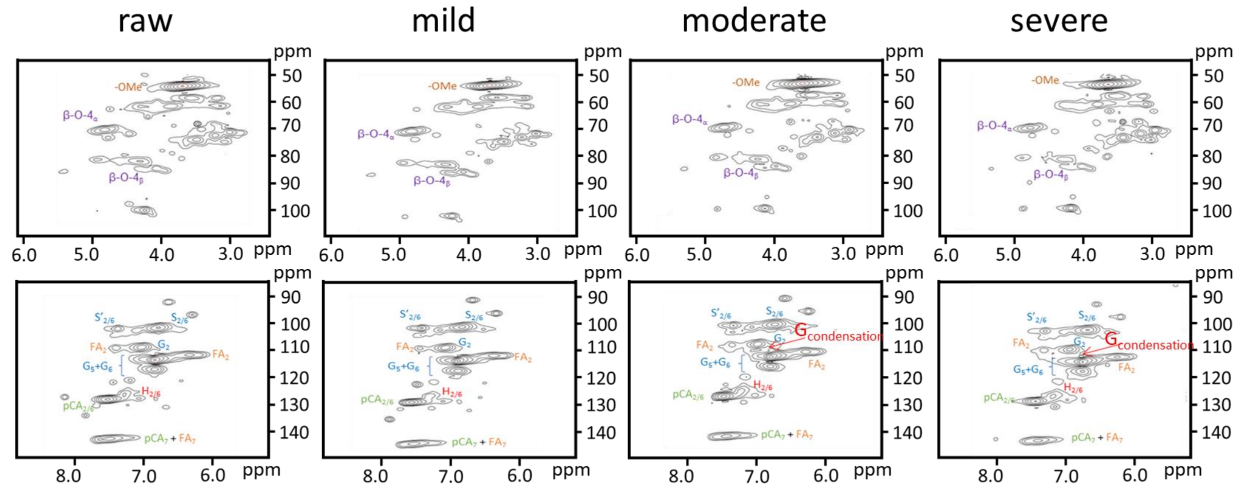

Figure S7. Comparison of HSQC NMR spectra of mill wood lignin. Lignin condensation was observed in moderate and severe storage conditions.

Table S1. Comparison of the 1600 cm^{-1} lignin aromatic Raman band intensity.

Sample Condition	Peak Area (cm^{-1})	Standard Deviation (cm^{-1})
Control	11.0	1.8
Mild	12.6	2.1
Moderate	11.5	2.5
Severe	11.9	2.9

Table S2. Comparison of the 1470 cm^{-1} xylan Raman band intensity.

Sample Condition	Peak Area (cm^{-1})	Standard Deviation (cm^{-1})
Control	15.4	3.0
Mild	14.1	3.2
Moderate	9.2	3.2
Severe	4.0	3.2

Table S3. Comparison of the 920 cm^{-1} xylan Raman band intensity.

Sample Condition	Peak Area (cm^{-1})	Standard Deviation (cm^{-1})
Control	14.8	2.8
Mild	16.2	2.9
Moderate	7.2	3
Severe	9.5	3.1

Table S4. Fitting results from log normal distribution function in Fig. S4.

	Control	Degraded
y0	-0.17569	9.8982E-4
xc	40.63123	9.70966
w	1.21018	0.72054
A	23.97371	5.02518
Reduced Chi-Sqr	5.17696E-4	7.56756E-4
R-Square (COD)	0.94903	0.96952
Adj. R-Square	0.9108	0.94667

REFERENCES

1. Ray, A. E.; Williams, C. L.; Hoover, A. N.; Li, C.; Sale, K. L.; Emerson, R. M.; Klinger, J.; Oksen, E.; Narani, A.; Yan, J.; Beavers, C. M.; Tanjore, D.; Yunes, M.; Bose, E.; Leal, J. H.; Bowen, J. L.; Wolfrum, E. J.; Resch, M. G.; Semelsberger, T. A.; Donohoe, B. S., Multiscale Characterization of Lignocellulosic Biomass Variability and Its Implications to Preprocessing and Conversion: a Case Study for Corn Stover. *ACS Sustainable Chemistry & Engineering* **2020,** *8* (8), 3218-3230.

2. Bose, E.; Leal, J. H.; Hoover, A. N.; Zeng, Y.; Li, C.; Ray, A. E.; Semelsberger, T. A.; Donohoe, B. S., Impacts of Biological Heating and Degradation during Bale Storage on the Surface Properties of Corn Stover. *ACS Sustainable Chemistry & Engineering* **2020,** *8* (37), 13973-13983.

3. Zeng, Y.; Zhao, S.; Wei, H.; Tucker, M.; Himmel, M.; Mosier, N.; Meilan, R.; Ding, S.-Y., In situ micro-spectroscopic investigation of lignin in poplar cell walls pretreated by maleic acid. *Biotechnol Biofuels* **2015,** *8* (1), 1-12.

4. Nam, S.; French, A. D.; Condon, B. D.; Concha, M., Segal crystallinity index revisited by the simulation of X-ray diffraction patterns of cotton cellulose Iβ and cellulose II. *Carbohydr Polymes* **2016,** *135*, 1-9.

5. Segal, L.; Creely, J. J.; Martin, A. E.; Conrad, C. M., An Empirical Method for Estimating the Degree of Crystallinity of Native Cellulose Using the X-Ray Diffractometer. *Textile Research Journal* **1959,** *29* (10), 786-794.

6. Kang, X.; Sun, Y.; Li, L.; Kong, X.; Yuan, Z., Improving methane production from anaerobic digestion of Pennisetum Hybrid by alkaline pretreatment. *Bioresource Technology* **2018,** *255*, 205-212.

7. Kang, X.; Zhang, Y.; Li, L.; Sun, Y.; Kong, X.; Yuan, Z., Enhanced methane production from anaerobic digestion of hybrid Pennisetum by selectively removing lignin with sodium chlorite. *Bioresource Technology* **2020,** *295*, 122289.

8. Thomas, J. C., The determination of log normal particle size distributions by dynamic light scattering. *Journal of Colloid and Interface Science* **1987,** *117* (1), 187-192.
